# Why Do Consumers Who Prefer Physical Entertainment Behave Digitally? Understanding the Preference–Behavior Gap

**DOI:** 10.3390/bs16050744

**Published:** 2026-05-11

**Authors:** Ștefan Bulboacă, Eliza Ciobanu, Ioana Bianca Chițu, Gabriel Brătucu, Cristinel Petrisor Constantin, Radu Constantin Lixăndroiu

**Affiliations:** 1 Department of Marketing, Tourism Services and International Affairs, Faculty of Economic Sciences and Business Administration, Transilvania University of Brașov, 500068 Brașov, Romania; stefan.bulboaca@unitbv.ro (Ș.B.); ioana.chitu@unitbv.ro (I.B.C.); gabriel.bratucu@unitbv.ro (G.B.); cristinel.constantin@unitbv.ro (C.P.C.); 2 Department of Management and Economic Informatics, Faculty of Economic Sciences and Business Administration, Transilvania University of Brașov, 500068 Brașov, Romania; lixi.radu@unitbv.ro

**Keywords:** preference–behavior gap, entertainment consumption, digital consumption, accessibility constraints, behavioral inconsistency, logistic regression

## Abstract

This study examines the discrepancy between stated preferences and actual behavior in entertainment consumption, with a particular focus on the role of perceived accessibility constraints. While literature suggests that factors such as time and cost may hinder the translation of preferences into behavior, the direction of this relationship remains unclear. Using survey data from 608 respondents, the analysis investigates why individuals who express a preference for physical entertainment often exhibit higher levels of digital consumption. To address this question, the study constructs composite indices capturing physical consumption (PCI), digital consumption (DCI), and perceived accessibility constraints (PACI), and estimates a series of logistic regression models to identify the determinants of the preference–behavior gap. The results reveal that perceived accessibility constraints have a statistically significant effect on behavioral inconsistency. However, contrary to initial expectations, the relationship is negative, indicating that higher perceived constraints are associated with a lower probability of exhibiting a preference–behavior gap. In contrast, gender differences remain significant, while consumption intensity measures do not explain the observed discrepancy. These findings suggest that the preference–behavior gap is shaped less by actual consumption patterns and more by how individuals perceive the feasibility of engaging in their preferred activities.

## 1. Introduction

While prior literature suggests that constraints such as time, cost, and effort may hinder the translation of preferences into actual behavior, this study tests whether perceived accessibility constraints increase the likelihood of a preference–behavior gap.

The literature on entertainment consumption shows that preferences are shaped by multiple motivations, including hedonic enjoyment, eudaimonic value, social interaction, curiosity, habit, and convenience. However, these motivations do not necessarily translate directly into behavior. Consumers may express preferences for entertainment forms that are meaningful, social, or physically immersive, while their actual consumption may be redirected toward digital alternatives that are easier to access, less costly, and more compatible with everyday routines. The entertainment offer has grown significantly recently through the development of digital platforms, which have become indispensable in meeting consumers’ needs for convenience (Kuriakose & Nagasubramaniyan, 2025). Digital entertainment can increase consumers’ interest, motivation, and the efficiency of physical training and can generate new development opportunities in physical preparation, providing consumers with a better training experience (Y. Zhang et al., 2025).

In this context, the main objective of the study is to identify the determinants of the preference–behavior gap in entertainment consumption, with a particular focus on the role of perceived accessibility constraints. More specifically, the analysis seeks to understand why individuals who express a preference for physical entertainment often display higher levels of actual engagement with digital alternatives. Accordingly, the study is guided by the following research question: Why do consumers who declare a preference for physical entertainment frequently behave in ways that are more strongly oriented toward digital consumption?

## 2. Literature Review and Theoretical Framework

### 2.1. Preference–Behavior Gap in Entertainment Consumption

The preference–behavior gap refers to situations in which individuals’ stated preferences do not fully correspond to their actual behavior. In consumer research, such gaps are often explained by the fact that preferences are shaped by attitudes, values, aspirations, and identity-related considerations, whereas behavior is also influenced by situational constraints, habits, convenience, and immediate availability. In entertainment consumption, this distinction is particularly relevant. Consumers may express preferences for physical entertainment because such activities are associated with social interaction, meaningful experiences, immersion, and emotional value. However, their actual behavior may be oriented toward digital entertainment because digital alternatives are easier to access, more flexible, and more compatible with everyday routines. Therefore, the preference–behavior gap in entertainment consumption reflects not only a difference between what consumers like and what they do, but also a broader tension between desired experiences and feasible actions.

The social context is particularly important in analyzing entertainment consumers’ experiences; these do not depend solely on the media content itself (Möller & Boukes, 2023) but also on the need to adapt content according to the mobile habits of generations, for example, Gen Z, whose motivations depend on perceptions of utility, lifestyle, entertainment, and convenience (the combination of news with entertainment) (Thi Nguyet Trang et al., 2025). Thus, market segmentation is recommended in order to adapt content to consumption behavior, even though information, positive emotions, and entertainment are the most sought-after gratifications (Gächter et al., 2025). Significant differences have been noted depending on age, gender, and the psychosocial characteristics of consumers (Menon, 2022).

Online consumer behavior depends on factors such as “positive value, self-brand congruity, social price, interaction control, human message interaction, informativeness, entertainment, and credibility” (Sun et al., 2024). For example, in the case of using Instagram reels, the following motivations were identified: “socially rewarding self-promotion, entertainment, escape, surveillance, novelty, documentation, and trendiness” (Menon, 2022). In the literature, the term “snack culture” has emerged, which refers to condensed and easily shareable content formats, increasingly popular at the expense of traditional film viewing. The facilitating factors are “perceived conciseness, entertainment value, ubiquity, trendiness of movie-condensed videos, and monetary cost of moviegoing significantly drove positive attitudes toward condensed videos” (Nguyen et al., 2025). Thus, snack culture and low-effort digital formats may help explain why consumers who value physical, or meaningful entertainment experiences nevertheless engage more frequently with digital content.

The study of intrinsic factors (related to individuals) as well as extrinsic factors (external actions) is addressed in many studies that examine entertainment consumption. For example, regarding e-sports, intrinsic motivation (pleasure) is what drives behavior, while extrinsic motivation (rewards, social pressure) has no significant effect (Tang et al., 2024). In entertainment streaming, user engagement is mainly driven by extrinsic motivation (rewards, social pressures, benefits); gamification and social sharing can stimulate engagement (Kao & Liu, 2025). The desire to learn or the challenge also appear as motivations in entertainment consumption; if applications are more challenging and arouse curiosity, people will be stimulated to use them in the future as well. Men are more motivated by challenge and curiosity, while women and people with higher incomes are more motivated by curiosity (Pham Thi & Duong, 2025). Motivation for entertainment is linked to FOMO (fear of missing out), which mediates the relationship between perceived stress and motivation for entertainment (Sever & Özdemir, 2021).

Most of the studies conducted to date related to the satisfactions obtained through digital games have been focused on the hedonic side (pleasure); however, recent research also recognizes eudaimonic experiences (deeper, for example, emotion, appreciation) (Possler et al., 2020). Thus, motivation for entertainment does not include only hedonic pleasure but also the “search for truth” (eudaimonic motivation) (Oliver & Raney, 2011).

In the field of digital entertainment, users seek significant eudaimonic experiences and the attainment of a sense of calm (Kuriakose & Nagasubramaniyan, 2025). The results of a study on online entertainment acceptance show the positive impact of habit and price value on behavioral intention, while social influence and hedonic motivation influence the formation of habit (Harnadi et al., 2025; Wohn & Ahmadi, 2019). This distinction is relevant for the present study because consumers may prefer entertainment experiences associated with deeper or more meaningful value, while engaging in digital alternatives that provide immediate gratification and require less effort.

A study conducted among adolescents revealed that, depending on their motivation for video games, they can be divided into 4 categories: role-players, fun-seekers, social gamers, and gaming maximizers; the classification was also influenced by levels of anxiety and sociability (David Bowman & Chang, 2023).

Motivation for participation in outdoor recreational activities—the experience shared with other people and the social aspects (belonging to a community) that contribute to well-being (Zwart & Hines, 2022). Experiencing video games fosters relationships and connections between generations, generating positive emotions for all participants; therefore, improving the social aspects of video games is necessary (Osmanovic & Pecchioni, 2016). Similarly, platform-driven mechanisms such as recommendation algorithms, rewards, and habitual use may shift actual behavior away from stated preferences, contributing to a preference–behavior gap.

At the same time, the social aspect, connection with other people, and belonging to a social group appear as motivations for entertainment consumption. The importance of building social relationships, as a motivation, not only for entertainment, online games are both a medium and a “result of the digital entertainment culture” (Ho, 2025).

The results of a study show that the consumption of cultural entertainment is based on emotional, cognitive, and social motivations such as entertainment, escapism, cultural exploration, learning/curiosity, and socialization (Manolika & Baltzis, 2020). The creation of digital cultural experiences should not be limited only to the faithful reproduction of physical appearance (authenticity), but should create deeper experiences for understanding context and emotional connection (S. Zhang et al., 2025).

Perceived economic mobility and social trust and social networks also influence entertainment consumption; individuals with good opportunities for economic growth and trust in others and in social networks are more motivated to participate in cultural activities (elite or popular) (Kim et al., 2025). In the case of gambling consumption, studies show that entertainment, rewards, and addiction to social networks directly and positively affect both consumption intention and the intention to share content (Ji et al., 2022).

### 2.2. The Role of the Perceived Accessibility Constraints

A useful theoretical lens for understanding this discrepancy is provided by Theory of Planned Behavior and other research on attitude–behavior inconsistency and perceived behavioral control. According to this perspective, preferences and intentions do not automatically translate into behavior when individuals perceive barriers that limit their ability to act (Ajzen, 2020). Constraints such as lack of time, financial costs, distance, limited availability, or effort requirements can reduce the perceived feasibility of engaging in preferred activities (de Matos et al., 2025; Elizondo-Candanedo et al., 2024). In the context of physical entertainment, accessibility constraints are especially relevant because participation often requires planning, mobility, financial resources, time allocation, and coordination with others. By contrast, digital entertainment usually requires less effort and is immediately available. As a result, consumers may continue to prefer physical entertainment while behaving digitally when perceived constraints reduce the likelihood of acting on those preferences (Chun et al., 2025; Ha & Kim, 2026; Leguina et al., 2025; Othman et al., 2024; Tutar & Turhan, 2023).

### 2.3. Hypothesis Development

Based on this theoretical reasoning, perceived accessibility constraints are expected to increase the likelihood of a preference–behavior gap. When individuals perceive stronger barriers to physical entertainment, the probability that their stated physical preference will not translate into actual physical consumption should increase. Instead, digital alternatives may function as more accessible substitutes.

Therefore, the study formulates the following hypothesis:

**H1.**  *Perceived accessibility constraints are positively associated with the likelihood of a preference–behavior gap in entertainment consumption.*

## 3. Materials and Methods

To examine the proposed relationship, the empirical strategy relies on a series of logistic regression models. The dependent variable captures whether individuals report a preference for physical entertainment while simultaneously exhibiting higher levels of digital consumption. This approach allows the study to move beyond descriptive patterns and provide a test of whether perceived accessibility constraints play a significant role in shaping the divergence between preferences and actual behavior.

### 3.1. Research Design

This study employs a quantitative explanatory design aimed at examining the determinants of the preference–behavior gap in entertainment consumption. The analysis focuses on the relationship between stated preferences for physical entertainment and actual behavior, measured through the relative intensity of digital versus physical consumption. The empirical strategy is centered on testing whether perceived accessibility constraints significantly influence the likelihood that a preference for physical entertainment does not translate into corresponding behavior.

### 3.2. Data and Sample

The empirical analysis is based on survey data collected through an online questionnaire. The full database contains 614 respondents, of whom 608 reported being interested in entertainment activities and were therefore retained as being eligible in the analytical sample. The final sample is appropriate for modeling entertainment-related consumer behavior, as it includes respondents with direct relevance to the phenomenon under investigation. The average age of the sample is 25 years, and the gender distribution is approximately balanced.

### 3.3. Data Preparation

The empirical analysis was conducted in several sequential steps. The first step consisted of retaining only respondents who declared interest in entertainment activities. This was implemented using the filter variable $V_{E}$ (interested by entertainment activities), where only cases with $V_{E}=1$ were included in the analysis. Secondly, three indices were constructed in order to operationalize the main concepts used in the model. To assess the internal consistency of the constructed measures, Cronbach’s alpha coefficients were calculated. The results indicate moderate levels of internal consistency for the consumption indices (PCI: α = 0.466; DCI: α = 0.574) and a higher level for the accessibility constraints index (PACI: α = 0.685). However, it is important to note that these indices are not conceptualized as reflective scales, but as formative constructs capturing heterogeneous components of behavior and perceived barriers. In the case of PCI and DCI, the indices aggregate different types of activities that are not expected to be highly correlated but represent distinct dimensions of entertainment consumption by reflecting the multidimensional and formative nature of the indices used in the analysis.

#### 3.3.1. Physical Consumption Index

Noted as PCI, this index captures the average intensity of physical entertainment consumption. It was computed as the arithmetic mean of the frequency variables:

(1) $${PCI}_{i}=\frac{1}{9}\sum_{j=1}^{9}{VEPA}_{i,j}$$

where ${VEPA}_{i,j}$ represents respondent i’s reported frequency of engagement in each physical entertainment activity.

#### 3.3.2. Digital Consumption Index

Digital Consumption Index, or DCI, captures the average intensity of digital entertainment consumption. It was calculated as:

(2) $${DCI}_{i}=\frac{1}{6}\sum_{k=1}^{6}{VDA}_{i,k}$$

where ${VDA}_{i,k}$ denotes the reported frequency of digital entertainment activities.

#### 3.3.3. Perceived Accessibility Constraints Index

Noted as PACI, the Perceived Accessibility Constraints Index, measures the average level of perceived barriers related to physical entertainment. It was constructed as:

(3) $${PACI}_{i}=\frac{1}{7}\sum_{k=1}^{7}{PPAB}_{i,m}$$

where ${PPAB}_{i,m}$ is the perceived presence of each accessibility-related barrier. This index measures the extent to which individuals perceive barriers to participating in physical entertainment activities. The index was constructed based on seven distinct accessibility-related constraints, including limitations related to time availability, financial cost, geographical distance, lack of companions, infrastructure, schedule constraints, and personal or to 0.86 (six out of seven barriers reported). The sample mean of 0.45 indicates that, on average, respondents perceive approximately three accessibility constraints. Moreover, given the bounded nature of the PACI index, the interpretation of the coefficient should be considered in terms of incremental changes rather than a full unit increase. More specifically, a 0.1 increase in the PACI index is associated with a decrease in the probability of exhibiting a preference–behavior gap. This interpretation provides a more realistic understanding of the effect size and highlights how incremental increases in perceived barriers influence behavioral consistency and contextual limitations. Each item was measured using a binary response format (1 = barrier present; 0 = barrier absent). As a result, the index ranges from 0 (no perceived barriers).

### 3.4. Dependent Variable

The dependent variable of the study is represented by a binary indicator capturing the preference–behavior gap. In this sense, the variable ${PrPh}_{i}$ which represents the preference for physical environment was created based on the survey variable as follows:

(4) $${PrPh}_{i}=\left\{\begin{array}{c} 1,\;if\;respondent\;i\;prefers\;physical\;entertainment\\ 0,\;otherwise \end{array}\right.$$

Therefore, the preference–behavior gap variable was defined as:

(5) $${GAP}_{i}=\left\{\begin{array}{c} 1,\;if\;the\;{PrPh}_{i}=1\;and\;{DCI}_{i}\;>{PC}_{i}\\ 0,\;otherwise \end{array}\right.$$

Thus, ${GAP}_{i}=1$ identifies respondents who state a preference for physical entertainment but whose actual consumption is more strongly oriented toward digital entertainment. To address the limitation of the binary classification, the authors constructed a continuous measure of the preference–behavior gap defined as the difference between digital and physical consumption intensity (DCI − PCI) for respondents who reported a preference for physical entertainment. This additional measure allows the analysis to capture the magnitude of the discrepancy and to distinguish between marginal and substantial gaps.

### 3.5. Independent Variables

PACI, is the focal explanatory variable of the study and is directly linked to the proposed hypothesis. Age, Gender, PCI and DCI are included as control variables. Age and gender account for socio-demographic differences, while PCI and DCI control for actual consumption intensity. These variables are included to isolate the effect of perceived accessibility constraints rather than to test separate hypotheses.

### 3.6. Econometric Model

Due to dependent variable, which is binary, the study uses binary logistic regression. The baseline model specification is as follows:

(6) $${Pr(GAP}_{i}=1)=\Lambda (\beta_{0}+\beta_{1}{PACI}_{i}{\;+\;\beta }_{2}A_{i}+\beta_{3}G_{i})$$

where Λ(·) is the logistic cumulative distribution function, A is age, and G is gender. To assess robustness, the model was extended as follows:

(7) $${Pr(GAP}_{i}=1)=\Lambda (\beta_{0}+\beta_{1}{PACI}_{i}{\;+\;\beta }_{2}{PCI}_{i}+\beta_{3}{DCI}_{i}+\beta_{4}A_{i}+\beta_{5}G_{i})$$

The final parsimonious model was specified to capture a final reduced specification retained only statistically significant predictors.

(8) $${Pr(GAP}_{i}=1)=\Lambda (\beta_{0}+\beta_{1}{PACI}_{i}{+\beta }_{2}G_{i})$$

### 3.7. Estimation Framework

**Figure 1:**
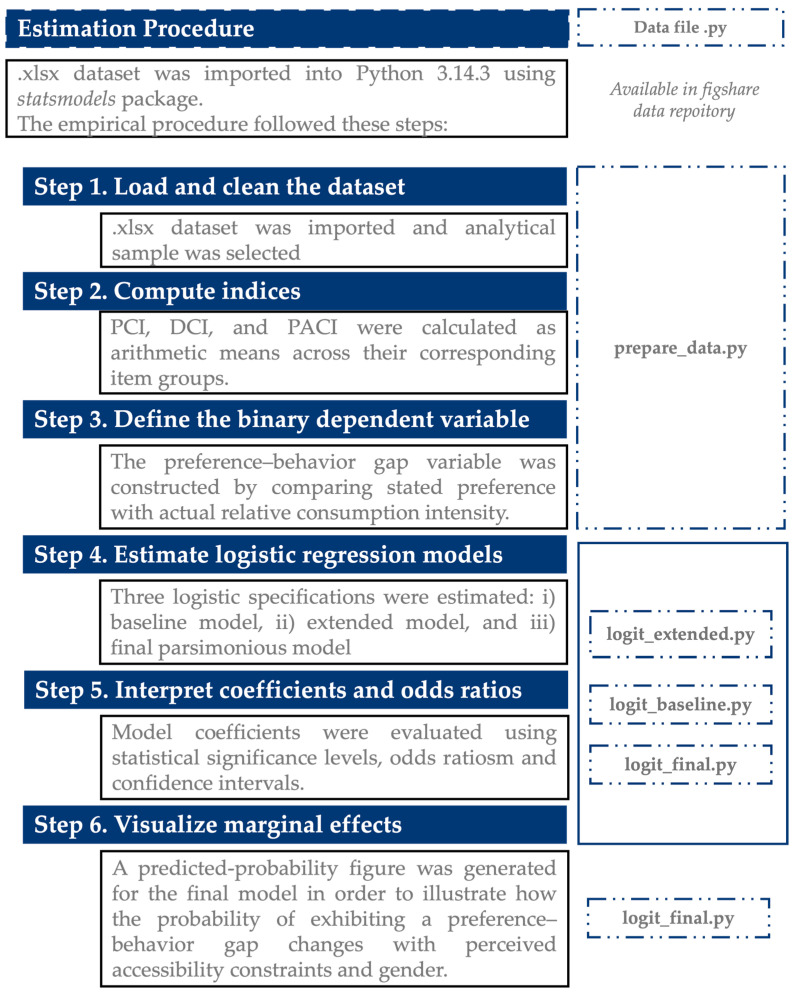
The estimation procedure. Source: authors conceptualization using Microsoft 365 PowerPoint 16.101.3.

Figure 1 illustrates the framework used after the data was collected, codified, and transposed into .xlsx file.

### 3.8. Methodological Argument

The choice of logistic regression is justified by the binary nature of the dependent variable. The use of composite indices allows the analysis to capture broader behavioral dimensions and not only relying on single survey items. In addition, the sequential estimation of baseline, extended, and parsimonious models improves robustness and allows the study to distinguish between the effects of perceived constraints and those of actual consumption intensity. The methodology is designed to test whether perceived accessibility constraints significantly influence the emergence of a discrepancy between stated preferences and observed entertainment behavior.

## 4. Results

### 4.1. Descriptive Statistics

Table 1 presents the main characteristics of the analytical sample, comprising 608 respondents who reported being interested in entertainment activities, ensuring relevance for modeling consumer behavior. The average age is 24.76 years (SD = 9.05), with a wide range between 18 and 70 years, indicating substantial heterogeneity within the sample. The gender distribution is balanced, with 50.3% female and 49.7% male respondents. In terms of occupational status, 52.6% of respondents are employed, suggesting a relatively active population. At general level, the sample provides a suitable basis for analyzing entertainment consumption behavior.

**Figure 2:**
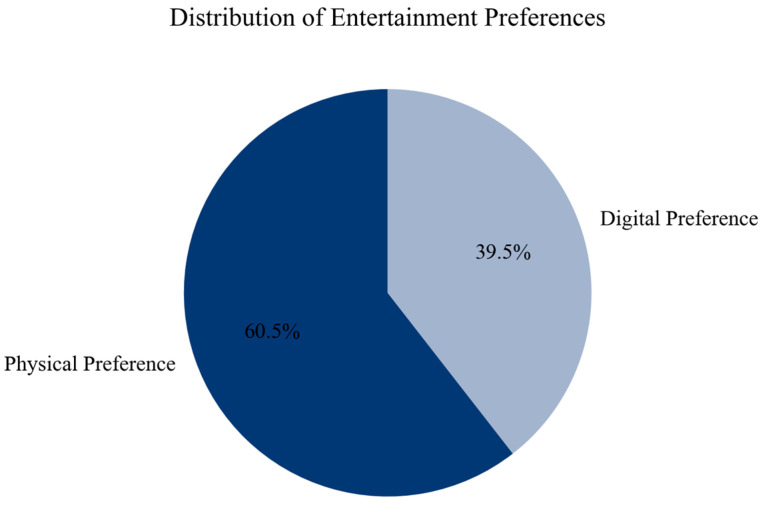
Distribution of entertainment preferences. Source: authors’ calculation based on primary data collection.

Figure 2 presents the distribution of stated preferences. A majority of respondents report a preference for physical entertainment, while 39.5% prefer digital alternatives. This indicates that physical entertainment remains dominant at the attitudinal level.

**Figure 3:**
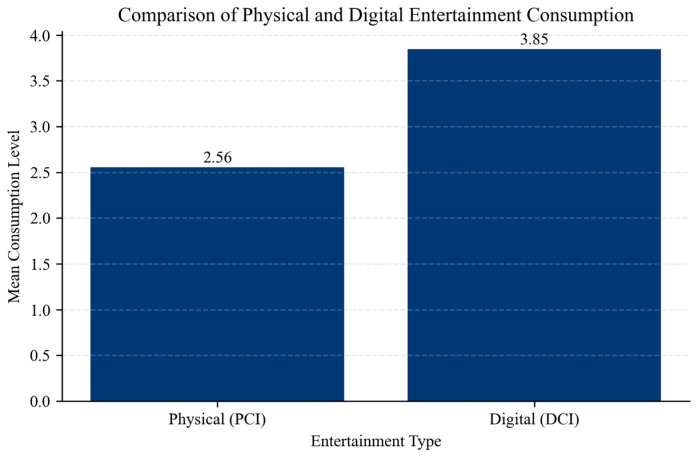
Physical and digital entertainment consumption. Source: authors’ calculation based on primary data collection.

Despite this preference structure, digital consumption levels are substantially higher than physical consumption, indicating a structural shift toward digital entertainment (Figure 3).

The descriptive statistics (Table 2) reveal a substantial difference between physical and digital consumption patterns. The Digital Consumption Index (DCI) has a significantly higher mean value (3.85) compared to the Physical Consumption Index (PCI) (2.56), indicating that digital entertainment dominates actual behavior. At the same time, the average level of perceived accessibility constraints (PACI) is moderate (0.45), suggesting that respondents experience barriers to physical participation, although with considerable variation across individuals.

**Figure 4:**
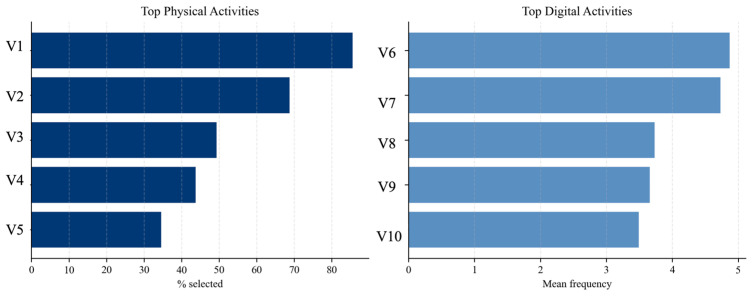
Top physical and digital entertainment activities. Source: authors’ calculation based on primary data collection. Note: V_n_ is the notation for activities.

To further explore the structure of entertainment consumption, Figure 4 presents the most frequently reported physical and digital activities. The results reveal that participation in physical activities is concentrated in a limited number of options, while digital consumption is more evenly distributed across activities. Moreover, the intensity of engagement with digital activities is consistently high, reinforcing the dominance of digital entertainment in actual behavior.

These findings reveal a clear preference–behavior paradox. Although most respondents express a preference for physical entertainment, their actual behavior is predominantly oriented toward digital activities. To further assess the magnitude of the preference–behavior gap, researchers examined the distribution of the difference between digital and physical consumption intensity among respondents classified as GAP = 1. This additional analysis allows us to distinguish between marginal, moderate, and substantial discrepancies. Table 3 shows that only 8.12% of the cases reflect marginal differences (DCI − PCI < 0.5), while 28.75% correspond to moderate discrepancies. Importantly, the majority of cases (63.12%) represent substantial gaps, where digital consumption significantly exceeds physical consumption. The average gap intensity is 1.30 (SD = 0.63), indicating that the discrepancy is not only widespread but also meaningful in magnitude. This output determines that the binary GAP variable captures a substantive behavioral phenomenon rather than being driven by marginal differences between digital and physical consumption.

**Figure 5:**
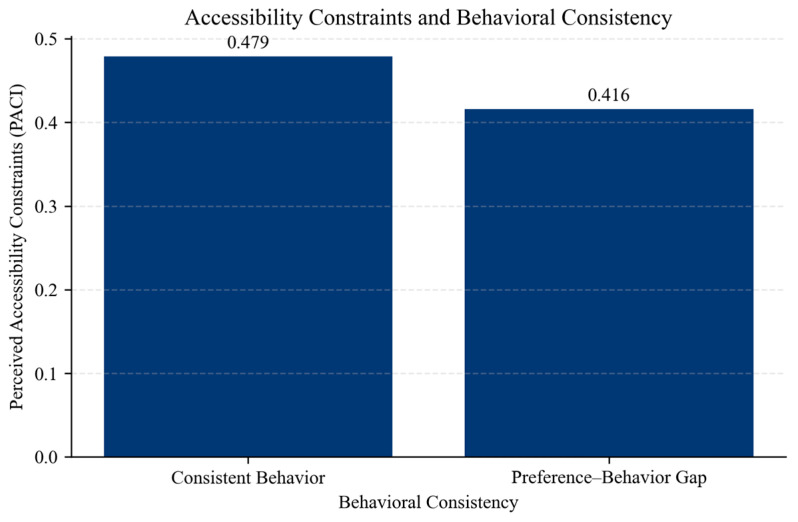
Accessibility constraints and behavioral consistency. Source: authors’ calculation based on primary data collection.

Furthermore, approximately 52.6% of respondents exhibit a preference–behavior gap, indicating that behavioral inconsistency is not a marginal phenomenon, but a dominant pattern within the sample. Figure 5 illustrates differences in perceived accessibility constraints across individuals with and without a preference–behavior gap. Individuals exhibiting a gap report lower perceived accessibility constraints compared to those with consistent behavior.

To further examine these differences, independent sample *t*-tests were conducted (Table 4). The results indicate that individuals exhibiting a preference–behavior gap report significantly lower levels of perceived accessibility constraints compared to those without a gap (*p* < 0.01). In contrast, differences in PCI, DCI, and age are not statistically significant.

These results suggest that behavioral inconsistency is not driven by differences in consumption intensity or demographic characteristics, but by the manner individuals perceive the feasibility of engaging in their preferred activities.

### 4.2. Empirical Results

Based on the descriptive findings, the authors highlight a substantial discrepancy between stated preferences and actual consumption patterns. They estimate a series of logistic regression models to formally examine the determinants of the preference–behavior gap. Table 5 presents the results of the baseline logistic regression model. The findings indicate that PACI have a negative and statistically significant effect on the likelihood of exhibiting a preference–behavior gap (*p* < 0.01). The associated odds ratio, below, suggests that higher perceived constraints substantially reduce the probability of behavioral inconsistency.

Table 6 reports the results of the extended model, which additionally controls for physical and digital consumption intensity (PCI and DCI). The inclusion of these variables does not alter the main findings. Perceived accessibility constraints remain negative and statistically significant (*p* < 0.01), confirming the robustness of the baseline results.

Neither PCI nor DCI exhibit statistically significant effects, suggesting that the preference–behavior gap is not driven by differences in consumption levels. Instead, the results indicate that behavioral inconsistency is more strongly associated with perceived feasibility rather than actual participation intensity.

Among the control variables, gender emerges as statistically significant, suggesting that the preference–behavior gap may vary across demographic groups. The effect is interpreted as an exploratory finding rather than as a separate relationship.

Table 7 presents the final parsimonious model, which retains only statistically significant predictors. The results confirm that perceived accessibility constraints and gender are the primary determinants of the preference–behavior gap.

To further evaluate the performance of the logistic regression model, several goodness-of-fit indicators were computed. The model yields a McFadden’s pseudo R^2^ of 0.027, indicating a modest but acceptable explanatory power, which is common in models of individual behavior (Appendix A). The AIC = 815.91 and BIC = 829.11 suggest that the model achieves a reasonable balance between fit and parsimony. In terms of predictive performance, the model achieves an overall classification accuracy of 58.5%. The sensitivity is relatively high (74.2%), indicating that the model performs well in identifying individuals who exhibit a preference–behavior gap. However, the specificity is lower (40.8%), suggesting that the model is less accurate in predicting cases without a gap. Overall, these results indicate that while the model captures key determinants of behavioral inconsistency, a substantial portion of the variation remains unexplained, pointing to the potential role of additional psychological or contextual factors not included in the analysis.

**Figure 6:**
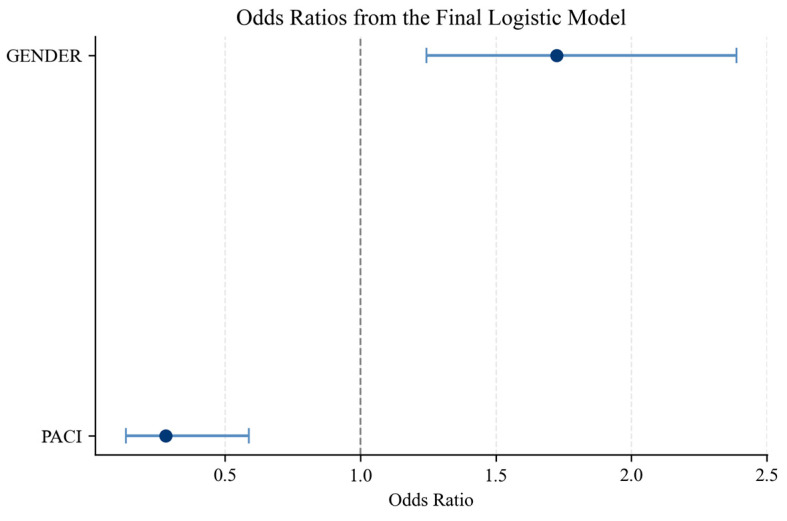
Estimated odds ratio (blue dots) plot. Source: authors’ calculation based on primary data collection.

To provide a more intuitive interpretation of the estimated effects, Figure 6 presents the odds ratios associated with the final logistic regression model. The graphical representation highlights both the direction and magnitude of the estimated relationships, as well as their statistical uncertainty.

The results confirm that perceived accessibility constraints have a strong negative effect on the likelihood of exhibiting a preference–behavior gap, as the associated odds ratio is substantially below one. In contrast, gender displays a positive effect, indicating that female respondents are more likely to exhibit behavioral inconsistency. The confidence intervals further confirm the statistical significance of both predictors.

Gender continues to have a positive and statistically significant effect. Female respondents are significantly more likely to exhibit a discrepancy between stated preferences and actual behavior, even after controlling for perceived accessibility constraints.

**Figure 7:**
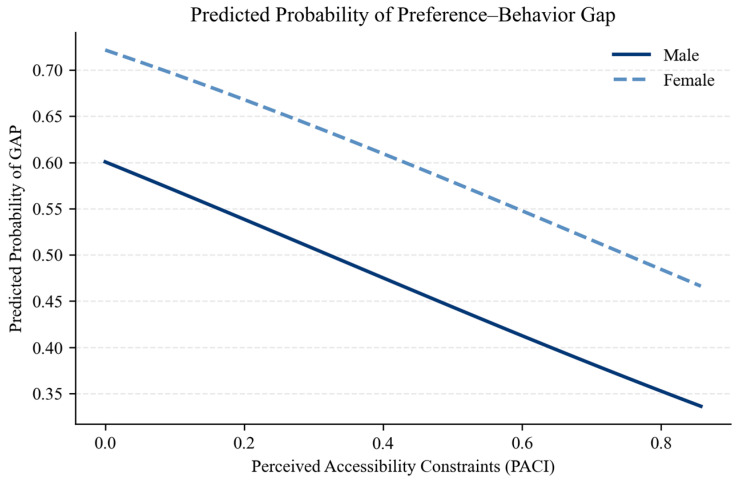
Predicted probability of preference–behavior gap. Source: authors’ calculation based on primary data collection.

Figure 7 provides a graphical representation of the marginal effects derived from the final logistic regression model. The results illustrate a clear negative relationship between perceived accessibility constraints and the probability of exhibiting a preference–behavior gap.

As illustrated in Figure 7, the predicted probability of exhibiting a preference–behavior gap decreases progressively as the number of perceived accessibility constraints increases, confirming the marginal effect of PACI across its observed range. In addition, the figure reveals a consistent gender gap. Across all levels of perceived accessibility constraints, female respondents display a higher predicted probability of exhibiting a preference–behavior gap compared to male respondents.

The results show that perceived accessibility constraints have a statistically significant effect on the preference–behavior gap. However, the direction of the relationship is negative, contrary to the theoretical expectation formulated in H1. Therefore, H1 is not supported in the expected direction.

## 5. Discussion

The findings of this study provide important insights into the preference–behavior gap in entertainment consumption by showing that perceived accessibility constraints significantly influence the likelihood of inconsistency between stated preferences and actual behavior. More specifically, the results indicate that individuals who declare a preference for physical entertainment may still engage more intensively in digital entertainment, and that this discrepancy is negatively associated with perceived accessibility constraints.

This result adds nuance to the existing literature on entertainment consumption, which generally emphasizes that time, cost, convenience, and accessibility shape how consumers translate motivations into actual behavior. Previous studies have shown that digital entertainment is strongly associated with convenience and ease of access, which makes it increasingly embedded in consumers’ everyday routines (Kuriakose & Nagasubramaniyan, 2025). Similarly, research on condensed digital content and mobile entertainment suggests that ubiquity, conciseness, and convenience are central drivers of positive attitudes toward digital alternatives (Nguyen et al., 2025). In this context, the present findings suggest that the preference–behavior gap may emerge not simply because consumers face barriers to physical entertainment, but because digital entertainment has become a highly practical and normalized substitute in everyday life.

At the same time, the negative sign of the coefficient for perceived accessibility constraints is particularly interesting. A conventional expectation would be that stronger perceived barriers increase the gap between what consumers prefer and what they do. However, the results indicate the opposite. Although H1 predicted a positive association between perceived accessibility constraints and the preference–behavior gap, the empirical results indicate a negative relationship. This suggests that higher perceived constraints do not widen the gap, but rather reduce behavioral inconsistency. One possible interpretation is that individuals facing stronger barriers adjust their preferences more realistically, while those facing fewer constraints may maintain aspirational preferences for physical entertainment but still default to digital behavior due to convenience, habit, or platform-driven accessibility. One possible interpretation is that consumers who perceive stronger accessibility barriers may also evaluate their possibilities more realistically and adjust their stated preferences accordingly. By contrast, individuals who perceive fewer barriers may continue to express a symbolic or aspirational preference for physical entertainment, while still choosing digital alternatives in practice because these are easier to integrate into daily routines. This interpretation is consistent with research showing that actual online behavior is shaped not only by utility, but also by entertainment, interaction control, informativeness, and credibility (Sun et al., 2024), as well as by habit and price value in online entertainment settings (Harnadi et al., 2025). In other words, behavior may be guided by the practical and habitual advantages of digital consumption even when attitudinal preferences remain oriented toward physical experiences.

The results also resonate with studies emphasizing that entertainment motivations are complex and multidimensional. Entertainment consumption is not determined by a single motive, but by a combination of hedonic, eudaimonic, social, and contextual factors (Oliver & Raney, 2011; Possler et al., 2020). Research has also shown that consumers seek not only pleasure, but also meaningful experiences, calm, curiosity, challenge, and social connection in digital environments (Kuriakose & Nagasubramaniyan, 2025; Pham Thi & Duong, 2025; Ho, 2025). Therefore, the fact that respondents report a preference for physical entertainment while behaving digitally may reflect the coexistence of different types of motivations rather than a simple contradiction. Physical entertainment may remain preferable at the symbolic or experiential level, while digital entertainment better satisfies routine needs related to accessibility, flexibility, and immediate gratification.

The findings are also broadly aligned with studies showing that the entertainment experience depends not only on the content itself, but also on the social and technological context in which it is consumed (Möller & Boukes, 2023). For younger and digitally immersed audiences, convenience and mobile compatibility are especially important in shaping engagement patterns (Thi Nguyet Trang et al., 2025). This helps explain why actual behavior may be more strongly oriented toward digital forms even when physical entertainment remains positively valued. In this sense, the preference–behavior gap identified in this study may reflect the growing hybridization of entertainment consumption, where attitudes are still attached to physical experiences, but behavior increasingly follows digital logic.

Another important result concerns gender. The analysis shows that female respondents are significantly more likely to exhibit a preference–behavior gap. This finding is consistent with prior literature indicating that entertainment motivations and media-related behavior vary by gender (Menon, 2022; Gächter et al., 2025; Pham Thi & Duong, 2025). Although the present study does not directly test the mechanisms behind this effect, the result suggests that the relationship between preference and behavior may be mediated by gender-specific patterns of motivation, routine formation, or contextual evaluation. This issue deserves further investigation in future studies.

By contrast, PCI, DCI, and age do not have significant effects in the regression models. This is also an important result. Existing literature often reports differences in entertainment motivation and media use across age groups (Menon, 2022; Harnadi et al., 2025), yet in the present study age does not explain the inconsistency between preference and behavior. Likewise, the lack of significance for PCI and DCI suggests that the gap is not merely a function of how much consumers participate in physical or digital entertainment, but rather of how they perceive and negotiate the feasibility of their preferred activity forms. This supports the view that behavioral inconsistency is more strongly linked to subjective interpretation and situational decision-making than to raw levels of consumption.

From a theoretical perspective, the study contributes to the literature by showing that stated preferences and actual behavior in entertainment consumption should not be assumed to converge automatically. Consistent with consumer behavior research emphasizing the role of contextual and motivational complexity, the present findings suggest that preferences may retain an aspirational, symbolic, or identity-related character, while behavior is shaped by convenience, accessibility, and habit. Thus, the preference–behavior gap should be understood as a distinct phenomenon in hybrid physical–digital consumption environments.

From a practical perspective, the findings imply that providers of physical entertainment should be cautious when relying exclusively on stated preference data. Consumers may report favorable attitudes toward physical entertainment yet still choose digital alternatives in practice. Given the strong role of convenience and habitual use identified in prior studies of digital consumption (Harnadi et al., 2025; Nguyen et al., 2025; Sun et al., 2024), managers should focus on reducing friction in the access and participation process and on making physical entertainment more compatible with everyday consumer routines.

The results show that perceived accessibility constraints have a statistically significant effect on the preference–behavior gap. However, the direction of the relationship is negative, contrary to the theoretical expectation formulated in H1. Therefore, H1 is not supported in the expected direction. By linking these results to previous studies on convenience, motivation, habit, and social context, the study provides a more nuanced explanation of why consumers who prefer physical entertainment often behave digitally.

## 6. Conclusions

The main contribution of this study lies in providing empirical evidence that challenges the conventional assumption that accessibility barriers necessarily increase the divergence between preferences and behavior. Instead, the results suggest that the preference–behavior gap in entertainment consumption is driven less by objective constraints and more by how individuals perceive and evaluate the feasibility of engaging in their preferred activities. By highlighting this inverse relationship, the study contributes to a more nuanced understanding of behavioral inconsistency in hybrid physical–digital consumption contexts.

From a theoretical perspective, the findings contribute to the literature on consumer behavior and entertainment consumption by demonstrating that stated preferences and actual behavior should not be treated as inherently aligned. The study extends existing research on motivation, accessibility, and digital consumption by showing that preferences may retain a symbolic or aspirational dimension, while behavior is primarily shaped by convenience, habit, and situational factors. In this sense, the preference–behavior gap emerges as a distinct phenomenon that reflects the coexistence of attitudinal attachment to physical experiences and the practical advantages of digital alternatives.

Furthermore, the results emphasize the importance of perceived feasibility as a key mechanism linking attitudes and behavior. This perspective complements existing frameworks that focus on motivations and constraints, suggesting that subjective interpretation of accessibility may play a more central role than previously assumed.

The findings have important implications for practitioners in the entertainment industry. First, providers of physical entertainment services should be cautious when relying solely on stated consumer preferences, as these may not accurately predict actual behavior. Even when consumers express a clear preference for physical experiences, they may still choose digital alternatives due to convenience, accessibility, and routine integration. Second, managers should focus on reducing friction in the consumption process by improving accessibility, flexibility, and ease of participation in physical entertainment. This may include simplifying booking processes, enhance location accessibility, or integrating digital elements into physical experiences to better align with consumer habits.

Finally, digital entertainment providers can benefit from the persistence of the preference–behavior gap, as it indicates that actual consumption is strongly influenced by convenience and habitual use, even among consumers who do not explicitly prefer digital formats.

Despite its contributions, the study has several limitations. First, the analysis is based on cross-sectional survey data, which limits the ability to establish causal relationships. Second, the sample is relatively young, with an average age of approximately 25 years, which may reduce the generalizability of the findings to older populations. Third, the use of self-reported measures may introduce response bias, particularly in the assessment of preferences and perceived constraints.

Additionally, the study relies on composite indices, which, while useful for capturing broader constructs, may obscure variation at the level of individual activities. Finally, the analysis does not explicitly incorporate contextual factors such as income, time availability, or geographic accessibility, which may also influence entertainment behavior.

Future research could address these limitations by using longitudinal data to better capture the dynamics between preferences and behavior over time. Experimental designs could also help establish causal mechanisms underlying the observed relationships. Moreover, future studies could explore additional moderating variables, such as income, lifestyle, urban versus rural context, or psychological traits, to better understand the heterogeneity of the preference–behavior gap. Qualitative approaches could also provide deeper insights into how consumers interpret accessibility constraints and how they negotiate trade-offs between physical and digital entertainment. Finally, given the rapid evolution of digital platforms, future research should further investigate how technological innovation, hybrid experiences, and platform ecosystems reshape the relationship between preferences and behavior in entertainment consumption.

## Figures and Tables

**Table 1:** Sample descriptive statistics.

Variable	Value
Sample size (N)	608
Mean age	24.76
Std. Dev. age	9.05
Age range	15–70
Female (%)	50.33
Male (%)	49.67
Employed (%)	52.63

Source: authors’ calculation based on primary data collection.

**Table 2:** Descriptive statistics of variables.

Variable	Mean	Std. Dev	Min	Max
Physical Consumption Index (PCI)	2.56	0.75	1.33	5.00
Digital Consumption Index (DCI)	3.85	0.59	2.20	5.00
Accessibility Constraints (PACI)	0.45	0.23	0.00	0.86
Age	24.76	9.05	15	70

Source: authors’ calculation based on primary data collection.

**Table 3:** The magnitude of the preference–behavior gap.

Gap Category	Frequency	Percentage
Marginal gap (0 < DCI − PCI < 0.5)	26	8.12%
Moderate gap (0.5 ≤ DCI − PCI < 1.0)	92	28.75%
Substantial gap (DCI − PCI ≥ 1.0)	202	63.12%

Source: authors’ calculation based on primary data collection.

**Table 4:** Group comparison and *t*-tests.

Variable	GAP	No GAP	t-Stat	*p*-Value
PACI	0.416	0.479	−3.462	0.0006
PCI	2.524	2.592	−1.079	0.281
DCI	3.822	3.877	−1.137	0.256
Age	24.97	24.54	0.585	0.559

Source: authors’ calculation based on primary data collection.

**Table 5:** Baseline logit.

Variable	Coefficient	Std. Eror	*p*-Value	Odds Ratio
Constant	0.550733	0.334859	0.100038	1.734524
PACI	−1.31313	0.385718	0.000663	0.268976
Age	−0.00514	0.009493	0.587972	0.99487
Gender	0.553423	0.167344	0.000943	1.739195

Source: authors’ calculation based on primary data collection.

**Table 6:** Extended logit.

Variable	Coefficient	Std. Eror	*p*-Value	Odds Ratio
Constant	1.326511	0.724972	0.067288	3.767874
PACI	−1.34147	0.397077	0.000729	0.261461
PCI	−0.16729	0.111735	0.134332	0.845951
DCI	−0.08064	0.150972	0.593261	0.922529
Age	−0.00647	0.009767	0.507851	0.993553
Gender	0.569416	0.168826	0.000744	1.767234

Source: authors’ calculation based on primary data collection.

**Table 7:** Final logit.

Variable	Coefficient	Std. Eror	*p*-Value	Odds Ratio
Constant	0.408	0.206	0.047	2.251
PACI	−1.269	0.377	0.001	0.588
Gender	0.544	0.166	0.001	2.389

Source: authors’ calculation based on primary data collection.

## Data Availability

All data are available in the Figshare data repository.

## Appendix A

**Table A1:** Model goodness of fit and predictive performance.

Indicator	Value
Log-Likelihood	−404.95
McFadden R^2^	0.027
AIC	815.91
BIC	829.11
Accuracy (%)	58.5
Sensitivity (%)	74.2
Specificity (%)	40.8

Source: authors’ calculation based on primary data collection. Note: Sensitivity represents the proportion of correctly predicted cases with a preference–behavior gap (GAP = 1), while specificity represents the proportion of correctly predicted cases without a gap (GAP = 0).
